# Thai Fruits Exhibit Antioxidant Activity and Induction of Antioxidant Enzymes in HEK-293 Cells

**DOI:** 10.1155/2016/6083136

**Published:** 2016-12-18

**Authors:** Natthinee Anantachoke, Pattamapan Lomarat, Wasin Praserttirachai, Ruksinee Khammanit, Supachoke Mangmool

**Affiliations:** ^1^Department of Pharmacognosy, Faculty of Pharmacy, Mahidol University, Bangkok 10400, Thailand; ^2^Department of Food Chemistry, Faculty of Pharmacy, Mahidol University, Bangkok 10400, Thailand; ^3^Department of Pharmacology, Faculty of Pharmacy, Mahidol University, Bangkok 10400, Thailand

## Abstract

The cellular antioxidant enzymes play the important role of protecting the cells and organisms from the oxidative damage. Natural antioxidants contained in fruits have attracted considerable interest because of their presumed safety and potential nutritional value. Even though antioxidant activities of many fruits have been reported, the effects of phytochemicals contained in fruits on the induction of antioxidant enzymes in the cells have not been fully defined. In this study, we showed that extracts from* Antidesma ghaesembilla*,* Averrhoa bilimbi*,* Malpighia glabra*,* Mangifera indica, Sandoricum koetjape*,* Syzygium malaccense,* and* Ziziphus jujuba *inhibited H_2_O_2_-induced intracellular reactive oxygen species production in HEK-293 cells. Additionally, these Thai fruit extracts increased the mRNA and protein expressions of antioxidant enzymes, catalase, glutathione peroxidase-1, and manganese superoxide dismutase. The consumption of Thai fruits rich in phenolic compounds may reduce the risk of oxidative stress.

## 1. Introduction

Oxidative stress is defined as an imbalance between endogenous antioxidant defense mechanisms and the production of reactive oxygen species (ROS), which at high levels can cause cell injury and damage through modifications of proteins, lipids, and DNA. Increased oxidative stress is involved in the pathophysiology of many diseases such as cardiovascular diseases [1], neurodegenerative diseases [2], and diabetes [3]. The cellular antioxidant enzymes play the important role of protecting the cells and organisms from the oxidative damage. For instance, superoxide dismutase (SOD) acts as a catalyst to convert superoxide radicals into oxygen and hydrogen peroxide [4]. The hydrogen peroxide converts into water and oxygen by catalase to protect the cells from accumulation of H_2_O_2_ [5]. Glutathione peroxidase (GPx) is the important enzyme to remove H_2_O_2_ by using glutathione as a substrate [6]. Heme oxygenase 1 (HO-1) is involved in antistress of physiological conditions including antioxidant and anti-inflammatory effects [7].

Epidemiological studies have found that consumption of fruits and vegetables has attracted growing interest because of their significant role in reducing the risk of cardiovascular diseases and other chronic diseases [8]. Consumption of antioxidants, through diet and supplements, is expected to remove ROS from the living system and provide health benefits. Several studies demonstrated that medicinal plants and fruits are a rich source of antioxidant compounds such as phenolics, flavonoids, quinones, vitamins, and alkaloids, which can decrease the incidence of oxidative stress and associated diseases [9–11]. Previous studies showed that the phytochemicals, especially phenolics, in fruits are the major bioactive compounds showing potent antioxidant effects [12–14]. There was a direct relationship between the total phenolic contents and the antioxidant activities in fruits [13–15].

Natural antioxidants that are presented in fruits have attracted considerable interest because of their presumed safety and potential nutritional and therapeutic value. The increased interest in natural antioxidants has led to the antioxidant evaluation of many species of fruits. Even though antioxidant activities of many fruits have been reported and the results showed that some of them could be rich sources of natural antioxidants, the effects of phytochemicals contained in fruits on the induction of antioxidant enzymes in the cells have not been fully defined. Therefore, the objectives of this study were to determine the profiles of total phenolics, total flavonoids, the antioxidant activities of Thai fruit extracts, and the molecular mechanisms of Thai fruit extracts on H_2_O_2_-induced oxidative stress in HEK-293 cells.

## 2. Materials and Methods

### 2.1. Chemicals

2,2-Diphenyl-1-picrylhydrazyl (DPPH), gallic acid, ascorbic acid, 6-hydroxy-2,5,7,8-tetramethylchroman-2-carboxylic acid (Trolox®), sodium nitrite, aluminium chloride hexahydrate, sodium hydroxide, hydrogen peroxide, 3-[4,5-dimethylthiazol-2-yl]-2,5-diphenyl tetrazolium bromide (MTT), and dichlorodihydrofluorescein (DCF) acetate were purchased from Sigma-Aldrich (Saint Louis, MO). Dulbecco's Modified Eagle Medium (DMEM), fetal bovine serum, 0.25% trypsin-EDTA solution, and penicillin-streptomycin solution were purchased from Gibco (Grand Island, NY). Folin–Ciocalteu's phenol reagent was purchased from Merck KGaA (Darmstadt, Germany). Anhydrous sodium carbonate was available from Ajax Finechem (Auckland, New Zealand).

### 2.2. Plant Materials

The fruits of* Antidesma ghaesembilla* Gaertn. (Phyllanthaceae),* Artocarpus integer* (Thumb.) Merr. (Moraceae),* Averrhoa bilimbi* L. (Oxalidaceae), and* Mangifera foetida* Lour. (Anacardiaceae) were collected from Ranong Province, Thailand, while the fruits of* Durio zibethinus* L. cultivar Mon Thong (Malvaceae),* Malpighia glabra* L. (Malpighiaceae),* Mangifera indica* L. cultivar Aok Rong (Anacardiaceae),* Musa paradisiaca* cultivar Awak (Musaceae),* Sandoricum koetjape* (Burm. f.) Merr. cultivar Tuptim (Meliaceae),* Syzygium malaccense* (L.) Merr. & Perry (Myrtaceae), and* Ziziphus jujuba* Mill. cultivar Milk Jujube (Rhamnaceae) were purchased from local markets in Bangkok, Thailand. The plants were identified by Professor Wongsatit Chuakul, the botanist and lecturer in the Department of Pharmaceutical Botany, Faculty of Pharmacy, Mahidol University.

### 2.3. Preparation of Extracts

Edible portions of the fresh fruits were run through a food chopper. The finely chopped fruits were dried by freeze dryer (FreeZone, Labconco). The coarsely ground dried fruits (50 g) were extracted with methanol (300–500 ml × 3 times). After filtration through filter paper (number 1, Whatman Inc.), the solvent was removed under reduced pressure using a rotary evaporator and the crude methanol extracts were stored at −20°C until analysis.

### 2.4. Determination of Total Phenolic Content

Total phenolic content of the fruit extracts was analyzed by the Folin–Ciocalteu colorimetric method described previously [16] with minor modifications. Stock solutions of the fruit extracts with concentration of 1.5 mg/ml were prepared in 50% methanol. Twenty microliters of the stock solutions was mixed with 50 *μ*l of 10% Folin–Ciocalteu reagent in 96-well plates and allowed to react for 3 min. The mixtures were then neutralized by adding 80 *μ*l of 7.5% of sodium carbonate solution. After incubation at room temperature for 2 h, the absorbance was recorded at 765 nm by an Infinite M200 Microplate Reader (Tecan). The assay was measured in triplicate and the results were calculated using a calibration curve for gallic acid (0.625 to 30 *μ*g/ml) and expressed as mg gallic acid equivalent (GAE) per 100 g fresh weight (mg GAE/100 g FW).

### 2.5. Determination of Total Flavonoid Content

Total flavonoid content was determined by aluminium chloride colorimetric test modified from previous study [17]. One hundred and sixty microliters of sterile water was added to the test tube followed by 40 *μ*l of the fruit extracts dissolved in sterile water at the concentration of 1 mg/ml and 15 *μ*l of 5% NaNO_2_. After 5 min, 24 *μ*l of 10% AlCl_3_·6H_2_O was added. The mixture was allowed to react for 6 min. Lastly, 80 *μ*l of 1 M NaOH and 80 *μ*l of sterile water were added and well mixed. Then 250 *μ*l of reaction mixture was transferred to 96-well plate. The plate was shaken for 30 sec and the absorbances were read at 510 nm by microplate reader. The total flavonoid content was expressed as quercetin equivalent per 100 g fresh weight (mg QE/100 g FW) which was calculated from the regression equation obtained from graph plotted between the absorbance and the concentration of quercetin at various concentrations (4–1,500 *μ*g/ml). The assay was performed in triplicate.

### 2.6. DPPH Radical-Scavenging Assay

Free radical-scavenging activity of the fruit extracts was determined based on the reduction of DPPH radicals by means of the modified spectrophotometric method in 96-well plates. The test samples were prepared at concentration of 2 mg/ml in methanol and mixed with 0.02% w/v DPPH radical methanolic solution 1 : 1 ratio in triplicate. The reaction mixtures were allowed to stand in the dark for 30 min at room temperature. The absorbance was measured at 517 nm using Infinite M200 Microplate Reader (Tecan). The DPPH radical-scavenging ability was calculated as percent inhibition by the following equation: %  Inhibition = [(*A*_control_ − *A*_sample_)/*A*_control_] × 100. The DPPH radical-scavenging activity of the fruit extracts was expressed as the IC_50 _values (*μ*g/ml) (inhibition of DPPH radical formation by 50% at concentration of 1 mg/ml). 6-Hydroxy-2,5,7,8-tetramethylchroman-2-carboxylic acid (Trolox) was used as a positive control.

### 2.7. Cell Culture

Human embryonic kidney-293 (HEK-293) cells were cultured using DMEM supplemented with 10% FBS and 1% (v/v) penicillin-streptomycin solution in a humidified atmosphere of 5% CO_2_ at 37°C as previously described [18]. Under serum starvation conditions, cells were treated with crude extract of Thai fruits.

### 2.8. MTT Assay

Cell viability was performed by the MTT assay according to the method previously described [19]. Cells were seeded in 96-well culture plates at a density of 1 × 10^4^ cells/well, in a total volume of 200 *μ*l of DMEM supplemented with 1% FBS plus 1% penicillin/streptomycin.

Cells were allowed to adhere to plate for 24 h, before being treated with solvent (vehicle control) and crude extracts at various concentrations (0.01–2000 *μ*g/ml) for 24 h. The experiments were performed in 2 replicate wells. The relative number of viable cells was then determined at 24 h after incubation, by adding 2 mg/ml of MTT solution and further incubating the cell for 4 h. The formazan crystals formed were then solubilized with DMSO. The plate was read using an Infinite M200 Microplate Reader (Tecan) at wavelength of 570 nm. The absorption value of the solution at 570 nm directly represents relative cell numbers. The percentage of cell viability was calculated according to the following equation:

(1) The % of cell viability=Absorbance of treated cellsAbsorbance of control cells×100.

### 2.9. Measurement of Intracellular ROS Level

The intracellular antioxidant activities of the crude extract of Thai fruits were quantified using a fluorescent probe dichlorodihydrofluorescein (DCF) diacetate (Sigma-Aldrich) to estimate the intracellular ROS production in the cell as previously described [20]. HEK-293 cells were seeded in a 12-well plate (5 × 10^4^ cells/well) or 35-mm glass bottomed dishes (1 × 10^5^ cells/dish) and treated with Thai fruit extracts (10 and 100 *μ*g/ml) for 12 h. After 12 h, the cells were then incubated with 100 *μ*M of H_2_O_2_ for 2 h and washed once with phosphate buffered saline (PBS), pH 7.4. Thereafter, 10 *μ*M DCFH-DA was added and incubated with the cells at 37°C in the dark for 30 min. The fluorescence intensity of DCF was determined using Multi-Detection Microplate Reader (BioTek Instruments) with an excitation wavelength of 485 nm and emission wavelength of 530 nm. To monitor the ROS production in living cells, HEK-293 cells were visualized using single-line excitation (488 nm) of an IX-81 fluorescence microscope (Olympus) with a 40x (NA 1.4) objective lens (Olympus).

### 2.10. mRNA Expression Analysis by RT-qPCR

The extraction of RNA was performed by using the RNeasy Mini Kit (Qiagen) as previously described [21]. RT-qPCRs for RNA expression were performed by using the KAPA SYBR FAST One-Step RT-qPCR Kit (KAPA biosystems) according to the manufacturer's instructions. RT-qPCRs were performed on Mx 3005p Real-Time PCR System (Stratagene). We designed and synthesized the primers as follows: human GPx-1 (sense, 5′-ctcttcgagaagtgcgaggt-3′; antisense, 5′-tcgatgtcaatggtctggaa-3′), human catalase (sense, 5′-gcagatacctgtgaactgtc-3′; antisense, 5′-gtagaatgtccgcacctgag-3′), human HO-1 (sense, 5′-caggcagagaatgctgag-3′; antisense, 5′-gcttcacatagcgctgca-3′), human Mn-SOD (sense, 5′-gcacattaacgcgcagatca-3′; antisense, 5′-agcctccagcaactctcctt-3′), human CuZn-SOD (sense, 5′-aaggccgtgtgcgtgaa-3′; antisense, 5′-caggtctccaacatgcctct-3′), human GRe (sense, 5′-cagtgggactcacggaagat-3′; antisense, 5′-ttcactgcaacagcaaaacc-3′), and human GAPDH (sense, 5′-cgagatccctccaaaatcaa-3′; antisense, 5′-gtcttctgggtggcagtgat-3′).

### 2.11. Western Blotting

Protein expression of antioxidant enzymes was performed by Western blotting as previously described [20]. Following stimulation, cells were washed once with PBS and solubilized in lysis buffer containing 20 mM Tris-HCl, pH 7.4, 0.8% Triton X-100, 150 mM NaCl, 2 mM EDTA, 10% glycerol, 100 *μ*M PMSF, 5 *μ*g/ml aprotinin, and 5 *μ*g/ml leupeptin. Protein concentration of cell lysates was determined using a Bio-Rad protein assay kit with bovine serum albumin as standard. Samples were mixed with loading buffer and denatured by heating at 95°C for 5 min prior to separation by SDS-PAGE. Separated proteins were transferred to polyvinylidene fluoride (PVDF) membrane (Bio-Rad) and subjected to immunoblotting with primary antibodies to catalase (Cell Signaling), Mn-SOD (Cell Signaling), GPx-1 (Abcam), and GAPDH (Cell Signaling). Immunoblots were visualized with HRP-conjugated secondary antibodies and a chemiluminescence detection system (GE Healthcare).

### 2.12. Statistical Analysis

Data were presented as mean ± SEM. The statistical analysis was determined using one-way analysis of variance (ANOVA) and Student's* t*-test, and values of *P* < 0.05 were considered to be significant.

## 3. **Results**

### 3.1. Yields of Thai Fruit Extracts

Eleven fruit samples were collected and bought from southern and central regions of Thailand and were authenticated by a botanist. The scientific names of the plants are shown in Table 1. Although there are many kinds of fruits in Thailand, in this study, we chose only 11 fruits that have a potential effect on inhibition of oxidative stress. Thai people consume significant amount of the investigated fruits at a ripe stage and the yields of the crude methanol extracts of Thai fruits ranged from 2.68 to 9.28% of fresh weight (edible part of fresh fruits weight) (Table 1).

### 3.2. Determination of Total Phenolic and Flavonoid Contents of Thai Fruit Extracts

The total phenolic contents were determined using the Folin–Ciocalteu method, a simple and widely used method, which relied on the transfer of electrons from phenolic compounds to the Folin–Ciocalteu reagent in alkaline medium. As shown in Table 1, the total phenolic contents of 11 selected Thai fruits are expressed as mg of gallic acid equivalent (GAE) per 100 g of fresh weight (FW). Among all the fruits analyzed, the highest phenolic content was found in* M. glabra* (723.83 ± 36.94 mg GAE/100 g FW), followed by* S. koetjape* (241.01 ± 16.51),* A. ghaesembilla* (98.38 ± 6.48),* M. indica* (45.39 ± 1.33),* Z. jujuba* (45.29 ± 3.45),* M. foetida* (38.83 ± 3.15),* A. bilimbi* (33.77 ± 1.37), and* S. malaccense* (29.55 ± 0.91) (Table 1). Phenolic content was slightly lower in* A. integer* and* D. zibethinus*.* M. paradisiaca* cultivar Awak had the lowest total phenolic content among the tested fruits (Table 1).

The total flavonoid contents of 11 selected Thai fruits are expressed as mg of quercetin equivalent (QE) per 100 g of fresh weight (mg QE/100 g FW). As shown in Table 1, total flavonoid contents of Thai fruit extracts ranged from 1.36 ± 1.67 to 851.49 ± 0.30 mg QE/100 g FW. The highest flavonoid content was found in* S. koetjape*, which had 851.49 ± 0.30 mg QE/100 g FW, followed by* M. glabra*,* A. integer*,* A. ghaesembilla*,* D. zibethinus*,* A. bilimbi*, and* M. foetida*, the values of which were 195.36 ± 0.14, 158.31 ± 1.02, 134.06 ± 2.90, 93.85 ± 0.36, 55.33 ± 0.70, and 46.13 ± 1.85 mg QE/100 g FW, respectively. Flavonoid content was lower in* S. malaccense* and* M. paradisiaca*. On the other hand,* Z. jujuba* had the lowest content of total flavonoid (1.36 ± 1.67), while that of* M. indica* was not detected.

### 3.3. DPPH Scavenging Activities of Thai Fruit Extracts

We next determined the antioxidant activity of fruit extracts using DPPH method. This assay based on the measurement of the antioxidant ability to scavenge the stable organic radical DPPH and used Trolox as positive control with the IC_50_ of 4.12 ± 0.3 *μ*g/ml. As shown in Table 1, the extract of* M. glabra* at the concentration of 1 mg/ml exhibited the highest DPPH free radical-scavenging activity with 96.62 ± 0.44% of inhibition (IC_50 _of 45.00 ± 1.90 *μ*g/ml), which related to the high total phenolic content (723.83 ± 36.94 mg GAE/100 g FW). The extracts of* S. koetjape* showed moderate DPPH scavenging activities 84.73 ± 1.38 (IC_50_ of 415.8 ± 1.5 *µ*g/ml), followed by* A. ghaesembilla* (67.65 ± 0.31; IC_50_ of 648.0 ± 6.2 *µ*g/ml),* M. indica* (44.85 ± 0.71), and* A. bilimbi* (30.66 ± 1.96). The extracts of* D. zibethinus*,* M. paradisiaca*, and* Z. jujuba* had DPPH scavenging activity less than 10% of inhibition (Table 1).

### 3.4. Correlation between Antioxidant Capacity and Total Phenolic Content

**Figure 1:**
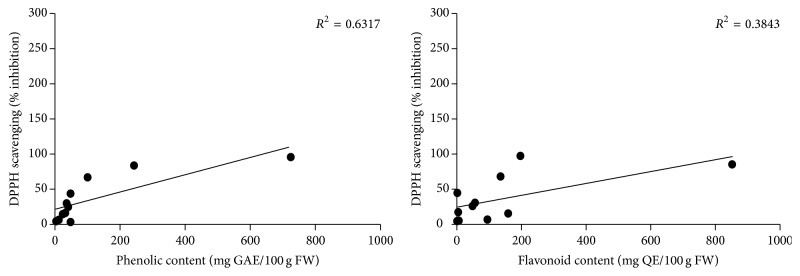
Correlation between antioxidant capacities and total phenolic and flavonoid contents in 11 Thai fruits. Antioxidant capacities of Thai fruit extracts were measured by the DPPH free radical-scavenging assay. GAE: gallic acid equivalent. QE: quercetin equivalent. FW: fresh weight.

The correlation between total phenolic content and antioxidant activity using DPPH assay of fruit extracts was determined and shown in Figure 1. A positive correlation (*R* ^2^ = 0.6317) between the DPPH scavenging value and total phenolic content indicated that phenolic compounds mainly contributed to the antioxidant activities of these fruits. This result was in agreement with several previous studies [15]. However, some fruits containing high total flavonoid compounds, such as* A. integer* and* D. zibethinus* (158.31 ± 1.02 and 93.85 ± 0.36 mg QE/100 g FW, resp.) did not exhibit strong antioxidant capacity. In contrast,* S. koetjape* and* M. glabra* that contained higher flavonoid contents elicited strong DPPH scavenging activity with 84.73 ± 1.38 and 96.62 ± 0.44% of inhibition, respectively. Thus, the correlations between antioxidant activity and total flavonoid contents of Thai fruit extracts were poor (*R* ^2^ = 0.3843) (Figure 1). According to their high antioxidants activities, it could be speculated that these fruits will be beneficial for inhibition of oxidative stress.

### 3.5. Thai Fruit Extract Inhibits H_2_O_2_-Induced Intracellular ROS Production in HEK-293 Cells

**Figure 2:**
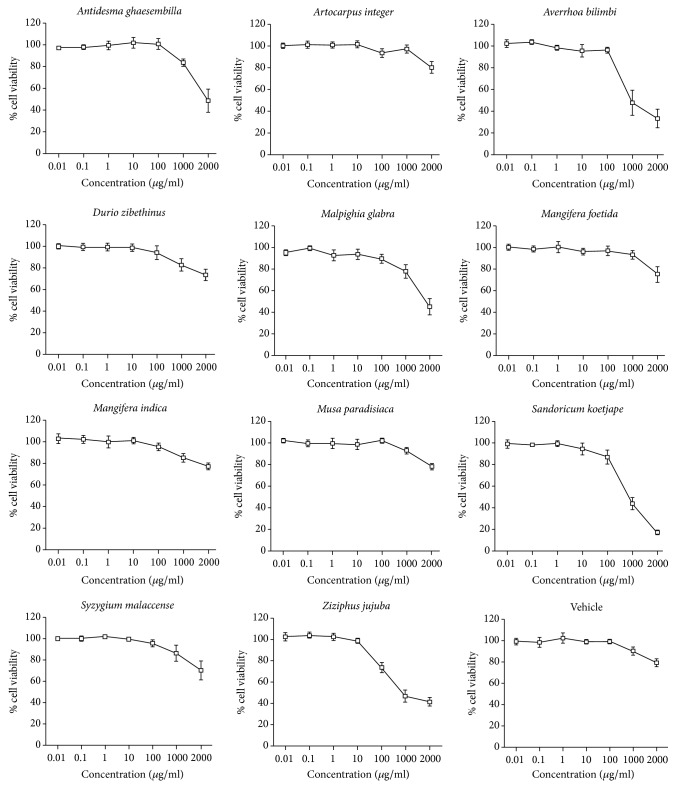
Cytotoxicity profile of Thai fruit extracts in HEK-293 cells. HEK-293 cells were treated with Thai fruit extracts (0.01–2,000 *μ*g/ml) for 36 h. Cell viability was quantified, expressed as a percentage of cell viability, and shown as the mean ± SEM (*n* = 4).

We first assessed the cell cytotoxicity of crude extracts of 11 Thai fruits on HEK-293 cells by using MTT assay to determine the optimal concentrations of the crude extract for measurement of intracellular antioxidant activity. We found that Thai fruit extracts at the concentration of 0.01–100 *μ*g/ml were not toxic to HEK-293 cells (Figure 2). Upon treatment of the cells with the crude extracts more than 100 *μ*g/ml, the cell viabilities of HEK-293 were less than 80%. The concentrations used in further experiment were 10 and 100 *μ*g/ml for the fruit extracts in order to prevent the cytotoxic effect and allow at least 80% cells survival.

**Figure 3:**
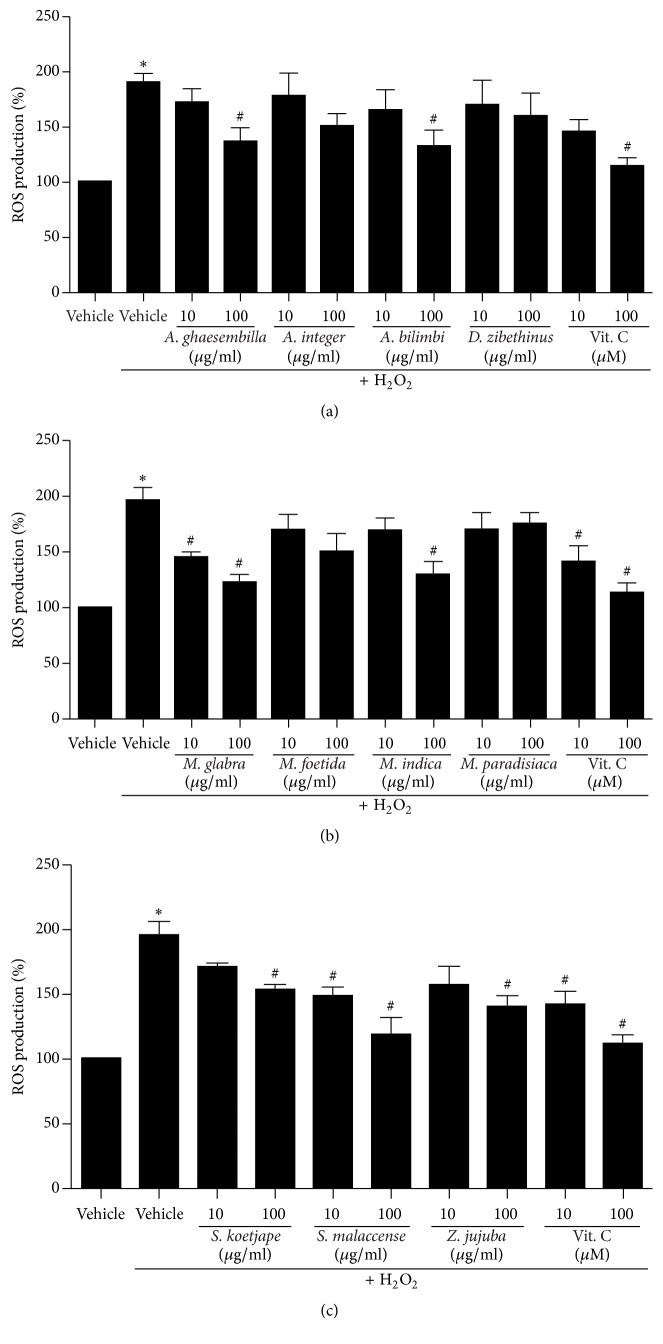
Effects of Thai fruit extracts on H_2_O_2_-induced ROS production in HEK-293 cells. (a–c) Cells were treated with vehicle (control), 10 and 100 *μ*M vitamin C, or 10 and 100 *μ*g/ml fruit extracts for 6 h. Cells were then incubated with 200 *μ*M H_2_O_2_ for 30 min. The intracellular ROS production was quantified, expressed as a percentage of the control, and shown as the mean ± SEM (*n* = 4). ^*∗*^ *P* < 0.05 versus control; ^#^ *P* < 0.05 versus H_2_O_2_.

We next investigated whether Thai fruit extract inhibits H_2_O_2_-induced intracellular ROS production in HEK-293 cells. The intracellular ROS levels were measured by using a fluorescent probe, DCFH-DA. After incubation with H_2_O_2_, the levels of intracellular ROS significantly increased compared to that of control (vehicle) group (Figure 3). As shown in Figure 3(a), treatment with* A. ghaesembilla* and* A. bilimbi* significantly inhibited H_2_O_2_-induced ROS production in a dose-dependent manner, whereas treatment with* A. integer* and* D. zibethinus* did not show the inhibitory effect on H_2_O_2_-induced ROS production. In addition, the crude extracts of* M. glabra* and* M. indica* showed a significant reduction in ROS production, which has effects similar to those of vitamin C (a potent antioxidant), whereas treatment with extracts of* M. foetida* and* M. paradisiaca* had no effect (Figure 3(b)). Moreover, treatment with fruit extracts of* S. koetjape*,* S. malaccense*, and* Z. jujuba* also suppressed H_2_O_2_-induced ROS production in a dose-dependent manner (Figure 3(c)).

**Figure 4:**
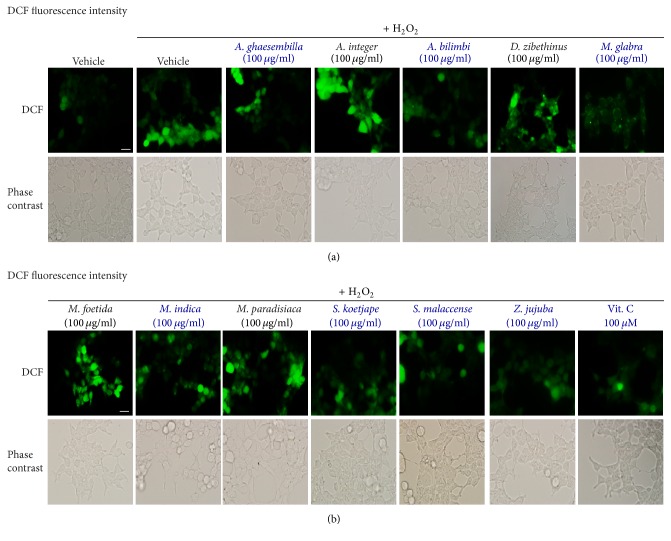
Thai fruit extracts attenuated intracellular ROS production in HEK-293 cells. (a-b) Cells were treated with vehicle (control), 10 and 100 *μ*M vitamin C, or 10 and 100 *μ*g/ml fruit extracts for 6 h. Cells were then incubated with 200 *μ*M H_2_O_2_ for 30 min. Cells were washed and incubated with 10 *μ*M DCFH-DA. Cells were visualized by green fluorescence for DCF and by bright field. Scale bar, 10 *μ*m (*n* = 4).

As shown in Figure 3, the fruit extracts of* A. ghaesembilla*,* A. bilimbi*,* M. glabra, M. indica, S. koetjape*,* S. malaccense, *and* Z. jujuba *have the antioxidant effects by preventing the production of intracellular ROS induced by H_2_O_2_. We further quantified ROS production in intact HEK-293 cells using the fluorescent microscope. Incubation of HEK-293 cells with H_2_O_2_ induced a significant increase in the fluorescence intensity of dichlorodihydrofluorescein (DCF) as shown in green color in intact cells of the control (vehicle) group (Figure 4). Treatment with fruit extracts of* A. ghaesembilla*,* A. bilimbi*,* M. glabra, M. indica, S. koetjape*,* S. malaccense, *and* Z. jujuba* decreased the fluorescence intensity of DCF, suggesting that these fruits suppressed the ROS production in intact cells. However, treatment with fruits extracts of* A. integer*,* D. zibethinus*,* M. foetida*, and* M. paradisiaca* did not reduce the fluorescence intensity of DCF inducing by H_2_O_2_ compared to that of control. Taken together, these data demonstrated that Thai fruits have the antioxidant effects by preventing the production of ROS in HEK-293 cells.

### 3.6. Induction of mRNA Expression of Antioxidant Enzymes by Thai Fruit Extracts

**Figure 5:**
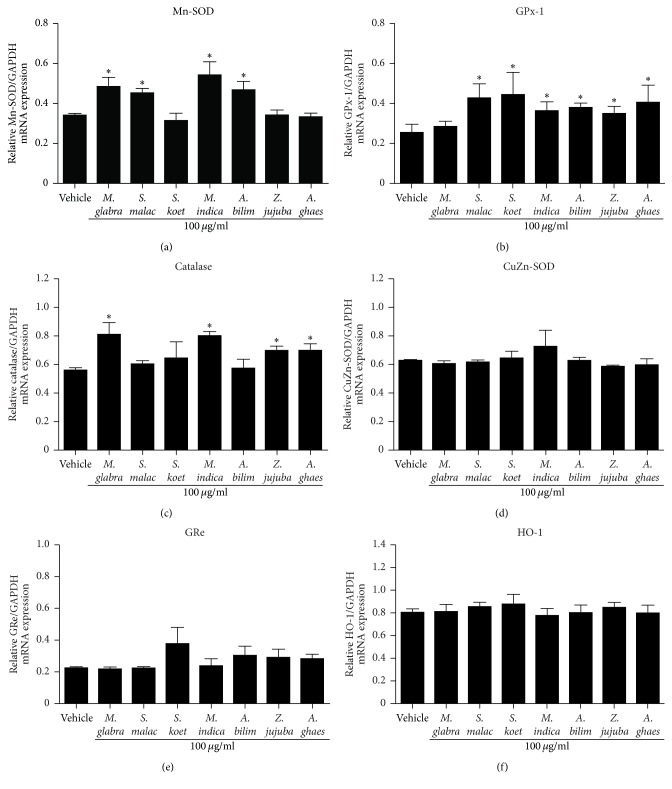
Effects of Thai fruit extracts on the mRNA expression of antioxidant enzymes. (a–f) Cells were treated with vehicle (control) or 100 *μ*g/ml fruit extracts for 6 h. After treatment, the total RNA was extracted from cells and the mRNA expression was analyzed using specific primers. The relative Mn-SOD (a), GPx-1 (b), catalase (c), CuZn-SOD (d), GRe (e), and HO-1 (f) mRNA levels were quantified and shown as the mean ± SEM (*n* = 4). ^*∗*^ *P* < 0.05 versus vehicle.

Antioxidant enzymes (e.g., GPx-1, catalase, Mn-SOD, CuZn-SOD, and HO-1) are the major components of antioxidant signaling cascade in many cell types. Treatment with* A. bilimbi* extract elicits the antioxidant effect by increasing the activity of SOD and GPx both in blood and in tissues (e.g., liver, kidney, and heart) of rats [22]. Moreover, an aqueous extract of* M. indica* inhibited oxidative stress by enhancing the production of antioxidant enzymes such as SOD and catalase [23]. Consistent with these previous studies, we showed that treatment with fruit extracts of* M. glabra*,* S. malaccense*,* M. indica,* and* A. bilimbi* significantly elevated the mRNA level of Mn-SOD (Figure 5(a)). Moreover, the GPx-1 mRNA expression significantly increased when treated with fruit extracts of* S. malaccense, S. koetjape, M. indica, A. bilimbi, Z. jujuba,* and* A. ghaesembilla* (Figure 5(b)). In addition, the mRNA level of catalase also increased after treatment with fruit extracts from* M glabra, M. indica, Z. jujuba,* and* A. ghaesembilla* (Figure 5(c)). However, all Thai fruit extracts failed to induce the mRNA expressions of CuZn-SOD, GRe, and HO-1 in HEK-293 cells (Figures 5(d)–5(f), resp.). Collectively our results showed that Thai fruit extracts play an important role in the prevention of oxidative stress by enhancing the mRNA expression of these antioxidant enzymes in HEK-293 cells.

### 3.7. Thai Fruit Extracts Increased Protein Level of Antioxidant Enzymes

**Figure 6:**
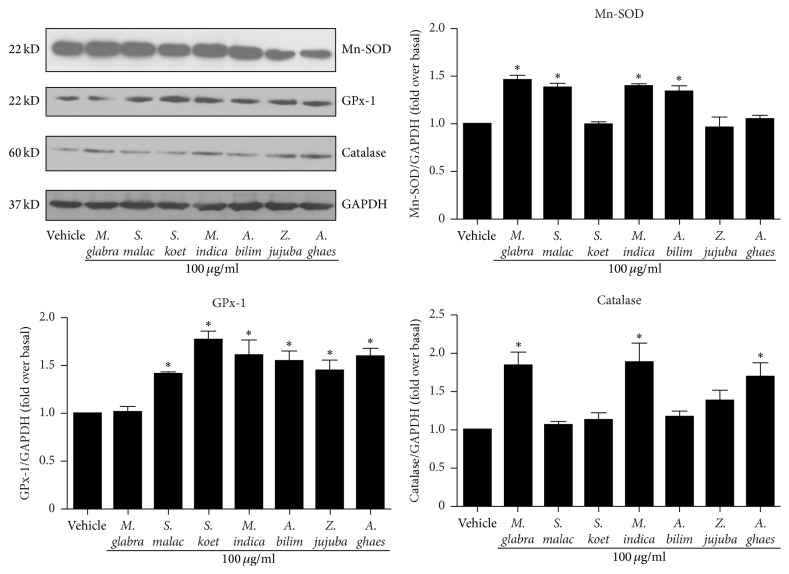
Effects of Thai fruit extracts on the protein expression of antioxidant enzymes. Cells were treated with vehicle (control) or 100 *μ*g/ml fruit extracts for 24 h. After treatment, the cell lysates were immunoblotted with anti-Mn-SOD, anti-GPx-1, or anti-catalase antibodies. The expression of GAPDH served as a loading control. The protein expression was quantified, expressed as the fold increase over vehicle (control) level, and shown as the mean ± SEM (*n* = 4). ^*∗*^ *P* < 0.05 versus control.

As shown in Figure 5, the mRNA expressions of Mn-SOD, GPx-1, and catalase were increased after treatment with the Thai fruit extracts. We next investigated the effects of these fruit extracts on the protein expressions of these antioxidant enzymes. We found that treatment with fruit extracts of* M. glabra, S. malaccense, M. indica,* and* A. bilimbi* at the concentration of 100 *μ*g/ml for 24 h significantly elevated the protein level of Mn-SOD (Figure 6, upper-right panel). Similar to the mRNA expression experiments, the results of protein expression revealed that treatment with fruit extracts of* S. malaccense, S. koetjape, M. indica, A. bilimbi, Z. jujuba,* and* A. ghaesembilla* caused an increase in the GPx-1 protein level compared with the control (vehicle) (Figure 6, lower-left panel). Moreover, the protein levels of catalase significantly increased after treatment with crude extracts of* M. glabra, M. indica,* and* A. ghaesembilla* for 24 h, whereas the catalase protein level tended to increase after treatment with crude extract of* Z. jujuba* (Figure 6, lower-right panel). These results indicated that Thai fruits prevent the oxidative stress by enhancing the protein synthesis of antioxidant enzymes.

## 4. Discussion

In this study, we provide a new molecular mechanism of Thai fruits extracts on the inhibition of oxidative stress in HEK-293 cells. We demonstrate that ethanol extracts from* A. ghaesembilla, A. bilimbi, M. glabra, M. indica, S. koetjape, S. malaccense,* and* Z. jujuba* have antioxidant effects by suppressing ROS production and inducing the synthesis of antioxidant enzymes such as GPx-1, catalase, and Mn-SOD in HEK-293 cells.

Oxidative stress has been implicated in the pathogenesis of various chronic diseases, such as cancer, heart diseases, and diabetes [1–3]. The nonenzymatic antioxidant defense system of the body is made up of some antioxidants, such as vitamin C, vitamin E, vitamin K, and glutathione. The exogenous antioxidants are mainly comprised of synthetic and natural antioxidants. Medicinal plants have been used to treat human diseases for thousands of years. People are becoming increasingly interested in medicinal plants, including fruits because of their good therapeutic performance and low toxicity. Fruits are potential sources of natural antioxidants and have been shown in epidemiological studies to be protective against several chronic diseases associated with aging such as cancer, immune dysfunction, and cardiovascular diseases [8]. These natural protective effects have been attributed to various components such as carotenoids, vitamins C and E, and phenolic compounds [24, 25]. Thus, the interest in phytochemicals including phenolic compounds derived from fruits and their roles in inhibition of oxidative stress are therefore increasing.

The antioxidative effects of Thai fruit extracts could be due to the presence of phenolic compounds. Studies have also shown that phenolic compounds from various sources are effective in improving antioxidant enzymes, suggesting their role in the prevention and treatment of oxidative stress-related diseases. Hence, the consumption of phenolic-rich fruits has been associated with reduced levels of ROS in animal experiments [10, 26]. Likewise, a high intake of phenolic-rich fruits has been reported to enhance the activities of the antioxidant enzymes [27]. It was reported that the antioxidant activity of raspberry and santol* (S. koetjape)* was directly related to the phenolic content [28]. In addition, the juice of* Ziziphus mauritiana* that has a moderate total phenolic content and low content of vitamin C elicits the antioxidant activity [29]. Consistent with these previous studies, our study demonstrated that the extract from* M. glabra* has the highest phenolic content, followed by* S. koetjape*,* A. ghaesembilla*,* M. indica*,* Z. jujuba*,* M. foetida*,* A. bilimbi*, and* S. malaccense* (Table 1). These Thai fruit extracts exhibited the antioxidant activity as determined by DPPH assay.

The correlation between total antioxidant capacities obtained from DPPH assay is shown in Figure 1. There was a direct linear relationship between the phenolic contents and total antioxidant activities in the tested 11 Thai fruits (*R* ^2^ = 0.6317). The higher total phenolic content in fruits resulted in higher total antioxidant activity. Similarly, previous study reported a significant positive correlation between the antioxidant capacity and the contents of total flavonoids and total phenolics in celery [30]. Moreover, antioxidant activities assayed by DPPH and FRAP methods showed strongly positive relationship with total phenolic content in Oxalidaceae fruits [31]. Taken together, these results suggested that phenolic compounds may be the major contributor to the total antioxidant activities of fruits. However, our results demonstrated a weak correlation (*R* ^2^ = 0.3843) between the DPPH scavenging activity and total flavonoid content suggesting that flavonoid compounds could not be main components responsible for DPPH scavenging activity of some fruits, such as* M. indica, S. koetjape,* and* M. glabra*.

In our present study, we found that, among the tested 11 Thai fruits tested, 7 of them showed the ability to inhibit H_2_O_2_-induced oxidative stress in HEK-293 cells. Previous study demonstrated that the administration of phenolic acids (i.e., gentisic acid, gallic acid, ferulic acid and* p*-coumaric acid) in rat at a concentration of 100 mg/kg exhibited an induction of hepatic antioxidant enzymes such as SOD and GPx [10]. This suggested that modulation of antioxidant enzymes and oxidative status in the liver by phenolic acids may play an important role in the protection against oxidative damage. Therefore, further identification of phytochemicals corresponded to antioxidant activities of Thai fruits are worth investigating.

Interestingly, the antioxidant effects of fruits might be due to the induction of antioxidant genes such as SOD and GPx [32, 33]. Fruit extracts from* Averrhoa carambola* (star fruit) also induced the activities of enzymatic antioxidants (i.e., SOD and catalase) and nonenzymatic antioxidant and reduced glutathione in mice [34]. Consistent with these previous studies, we showed that treatment with the Thai fruit extracts from* M. glabra*,* S. malaccense*,* M. indica,* and* A. bilimbi* induced the mRNA and protein expressions of Mn-SOD. We also demonstrated that fruit extracts from* M. glabra, M. indica,* and* A. ghaesembilla* are able to induce the mRNA and protein expressions of other antioxidant enzymes such as catalase. However, our study is unlike the study by others in which activities of these antioxidant enzymes were not determined after treatment with fruit extracts in HEK-293 cells. Further evaluation of the Thai fruit extracts-induced antioxidant enzyme activity in HEK-293 cells is required.

**Figure 7:**
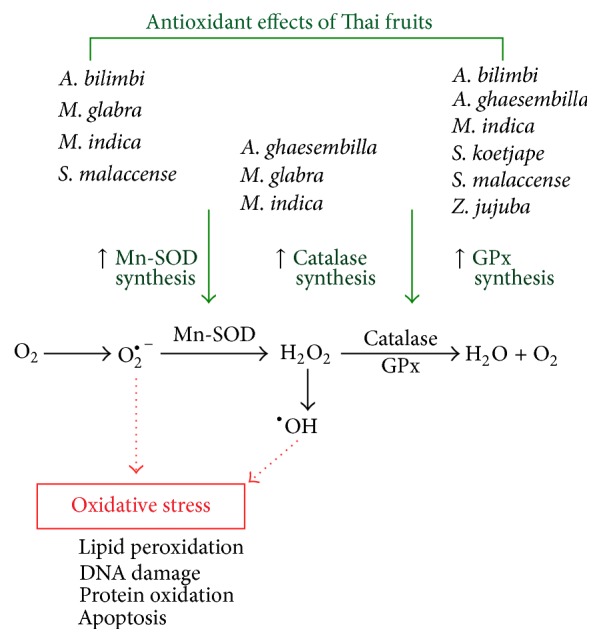
Schematic diagram representing the antioxidant effects of Thai fruits in HEK-293 cells. Thai fruit extracts from* Antidesma ghaesembilla*,* Averrhoa bilimbi*,* Malpighia glabra*,* Mangifera indica, Sandoricum koetjape*,* Syzygium malaccense,* and* Ziziphus jujuba *inhibit oxidative stress in HEK-293 cells by increasing the production of antioxidant enzymes such as catalase, GPx, and Mn-SOD.

There are two categories of antioxidative defenses that prevent radical formation, repair oxidative damage, remove radicals before damage can occur, eliminate damaged molecules, and prevent mutations. These two categories are antioxidant enzymes and nonenzymatic antioxidant compounds [35]. SOD, catalase, and GPx are enzymes that destroy the peroxides and play a significant role in providing antioxidant defenses to an organism. GPx and catalase are involved in the elimination of H_2_O_2_. SOD acts by dismutating superoxide radical to H_2_O_2_, which is then acted upon by GPx (Figure 7). The functions of all three enzymes are interconnected and their insufficient activities result in the accumulation of ROS production and increased oxidative stress in the cells.

The previous study suggested that treatment with the aqueous extract of* A. bilimbi* can partially reduce the imbalance between the ROS generation and the scavenging enzyme activity in diabetic animals exposed to oxidative stress [33]. Consistent with this previous study, we demonstrated that* A. bilimbi* extract is able to inhibit H_2_O_2_-induced ROS production in HEK-293 cells by inducing the synthesis of antioxidant enzymes such as Mn-SOD and GPx-1. According to this previous study and our present data,* A. bilimbi *could be a supplement, as an antioxidant therapy, and may be beneficial for correcting the ROS production and preventing oxidative stress due to lipid peroxidation and free radicals.

It has been reported that mango peel extract contains several valuable compounds such as polyphenols, carotenoids, terpenoids, sterols, fatty acids, and dietary fiber [12]. The major components such as mangiferin, amentoflavone, friedelin, daucosterol, and beta-sitosterol have been already identified [36]. The peel extract of* M. indica* exhibited high antioxidant capacity by effectively scavenging various free radical such as DPPH, hydroxyl, and peroxyl radicals [12]. In addition, treatment with aqueous extract of* M. indica* inhibited lipofundin-induced oxidative stress in rats [23]. This antioxidant effect is derived from the increasing activities of SOD and catalase [23]. We also showed in this study that* M. indica* extract has an antioxidant activity* in vitro* and in HEK-239 cells. This extract induced the mRNA and protein expressions of Mn-SOD, GPx-1, and catalase in HEK-293 cells. Thus, the active compounds in mango extract might exert the protective effects through stimulating oxidative defense system in the cells.

*Malpighia glabra* (Escobillo) is well known as an excellent dietary source of vitamin C and many phytochemicals such as polyphenols, anthocyanin, carotenoids, and tannins [37]. Interestingly, the phytochemical estimation in ten Indian fruits revealed that* M. glabra* has the highest phenolic content in methanol extract (355.74 ± 4.29 mg GAE/100 g FW) [37]. Our data are consistent with this previous study showing that, among 11 fruits, the highest phenolic content was found in* M. glabra* (723.83 ± 36.94 mg GAE/100 g FW). The difference in phenolic content may be due to the variation in the state of maturity, genotype, and climatic factors. Previous study reported that aqueous extract of* M. glabra* has a potent antioxidant activity determined by DPPH scavenging test [38]. In addition, our present study reported that treatment with extract from* M. glabra* attenuated H_2_O_2_-induced ROS production in HEK-293 cells, confirming that the fruits of* M. glabra* are rich in antioxidants especially polyphenols. The study from Singh and his colleagues showed the positive correlation for antioxidant capacity with phenolic compounds (*R* ^2^ = 0.845) [37]. We also reported the strong correlation between antioxidant capacity and phenolic contents in Thai fruits including* M. glabra*. In addition, we found that the methanol extract of* M. glabra* exhibited the antioxidant effect in HEK-293 cells by increasing mRNA and protein expressions of Mn-SOD and catalase. Hence, the fruits of* M. glabra* could be a good resource of phytochemicals with high antioxidant activity. However, further studies on the identification and isolation of individual active compounds are required.

*A. ghaesembilla* is an indigenous species found in Thailand. A few observations demonstrated that the extract of* A. ghaesembilla* has antioxidant activity. For instance,* A. ghaesembilla* was analyzed for their total antioxidant capacity through* in vitro* radical-scavenging assays such as DPPH and FRAP assays and antioxidant enzyme activity assay [32]. This previous study reported that extract from* A. ghaesembilla* has the highest DPPH (1,020.6 AEAC mg/100 g dry wt.) and FRAP values (2,114 *μ*M AEAC/g dry wt.). In addition,* A. ghaesembilla* can activate the enzymatic activities of SOD, catalase, and peroxidase [32]. In the present study, we reported that the extract of* A. ghaesembilla* at the concentration of 1 mg/ml exhibited moderate DPPH free radical-scavenging activity with 67.65 ± 0.31% of inhibition, which related to the total phenolic content (98.38 ± 6.48 mg GAE/100 g FW). Moreover, the extract of* A. ghaesembilla* showed an antioxidant effect in HEK-293 cells by stimulating the synthesis of GPx-1 and catalase. Interestingly, administration of crude extract (dose 400–1,000 mg/kg body weight) from* A. ghaesembilla* for 21 days in diabetic rats resulted in a decrease of blood glucose level [39]. As regards antioxidant and hypoglycemic actions,* A. ghaesembilla* can be served as a good source of natural supplement.

The juice of* Z. mauritiana* has moderate total phenolic content (396.96 ± 7.77 *μ*g GAE/g sample) while juice of* S. malaccense* has low total phenolic content (81.51 ± 0.06 *μ*g GAE/g sample) [29]. Consistent with this previous study, we reported that fruit extract of* Z. jujuba* exhibited higher phenolic content than* S. malaccense* (45.29 ± 3.45 versus 29.55 ± 0.91 mg GAE/100 g FW). The juice of* Z. mauritiana *and* S. malaccense* exhibited the great DPPH scavenging activity (460.58 ± 17.93 and 137.11 ± 2.07 *μ*g VCEAC/g sample, resp.) [29]. In addition, our study showed that the methanol extracts of* Z. jujuba *and* S. malaccense* fruits also exhibited the DPPH scavenging activity. Moreover, these two fruits attenuated H_2_O_2_-induced ROS production and increased the synthesis of GPx-1 and catalase in HEK-293 cells. The antioxidant activity of* Z. mauritiana* might be contributed by* p*-hydroxybenzoic acid that was reported to be the most abundant phenolic compound in the fruit extract [40].

The juice of* S. koetjape* has high total phenolic content (617.04 ± 2.23 *μ*g GAE/g sample) [29]. In addition, we reported that higher phenolic content was also detected in crude methanol extract of* S. koetjape* (241.01 ± 16.51 mg GAE/100 g FW). The antioxidant activities of* S. koetjape* could be associated with its high total phenolic content. The juice of* S. koetjape* exhibited the great DPPH scavenging activity (418.22 ± 5.17 *μ*g VCEAC/g sample) [29]. Consistent with this previous study, we found that methanol extract of* S. koetjape* showed a high DPPH scavenging activity (Table 1). Moreover,* S. koetjape* extract elicited the antioxidant activity in HEK-293 cells by attenuating H_2_O_2_-induced ROS production. Our present study demonstrated that the antioxidant activity of* S. koetjape* extract derived from the induction of mRNA and protein expression of GPx-1 in HEK-293 cells.

It has been reported that administration of phenolic acids (i.e., gentisic acid, gallic acid, ferulic acid, and* p*-coumaric acid) at a dosage of 100 mg/kg for 14 consecutive days in rats enhanced the mRNA expression of hepatic CuZn-SOD, GPx, and catalase [10]. Moreover, these phenolic acids also stimulated the enzyme activities. In the present study, we demonstrated that Thai fruits that have high phenolic contents showed the antioxidant effects by increasing the synthesis of Mn-SOD, GPx-1, and catalase in HEK-293 cells. Thus, polyphenolics found in fruits might exert the antioxidant effect through stimulating synthesis of these antioxidant enzymes. Although it is possible that phenolic acids contained in Thai fruits are involved in the regulation of antioxidant enzyme synthesis, further study is required to determine the active compounds necessary for antioxidant actions of Thai fruits in HEK-293 cells.

## 5. Conclusion

We have identified a new mechanism for fruit extracts mediating the inhibition of oxidative stress in HEK-293 cells. Thai fruits provide an antioxidation, which enhances the mRNA and protein expressions of antioxidant enzymes such as catalase, GPx-1, and Mn-SOD (Figure 7). Our results provide a better understanding of the antioxidant effects of Thai fruits on antioxidant enzyme production in the cells as well as information regarding the intake of fruits for human health.

## Figures and Tables

**Table 1:** Yield of methanol extracts, total phenolic and flavonoid contents, and DPPH scavenging activities of Thai fruit extracts.

Fruits (ripe stage)	Scientific name	Yield of crude extract (% FW)	Total phenolic content (mg GAE/100 g FW)	Total flavonoid content (mg QE/100 g FW)	DPPH scavenging activity (% inhibition at 1 mg/mL)
Mao khai pla (Thai)	*Antidesma ghaesembilla* Gaertn.	4.83	98.38 ± 6.48	134.06 ± 2.90	67.65 ± 0.31
Champedak	*Artocarpus integer* (Thumb.) Merr.	6.85	21.45 ± 2.28	158.31 ± 1.02	15.42 ± 1.52
Bilimbi	*Averrhoa bilimbi* L.	2.68	33.77 ± 1.37	55.33 ± 0.70	30.66 ± 1.96
Durian	*Durio zibethinus* L. cultivar Mon Thong	4.86	9.65 ± 0.71	93.85 ± 0.36	6.80 ± 0.97
Escobillo	*Malpighia glabra* L.	4.40	723.83 ± 36.94	195.36 ± 0.14	96.62 ± 0.44
Horse mango	*Mangifera foetida* Lour.	6.74	38.83 ± 3.15	46.13 ± 1.85	25.63 ± 1.32
Mango	*Mangifera indica* L. cultivar Aok Rong	9.12	45.39 ± 1.33	ND	44.85 ± 0.71
Pisang Awak	*Musa paradisiaca *L. cultivar Awak	7.24	0.34 ± 0.15	3.70 ± 1.31	5.15 ± 1.15
Santol	*Sandoricum koetjape* (Burm. f.) Merrill cultivar Tuptim	9.28	241.01 ± 16.51	851.49 ± 0.30	84.73 ± 1.38
Malay apple, Pomerac	*Syzygium malaccense* (L.) Merr. & Perry	4.28	29.55 ± 0.91	3.99 ± 1.65	16.75 ± 1.08
Jujube	*Ziziphus jujuba* Mill. cultivar Milk Jujube	7.63	45.29 ± 3.45	1.36 ± 1.67	4.45 ± 0.91

ND = not detected.
